# Electrochemical Analysis of Curcumin in Real Samples Using Intelligent Materials

**DOI:** 10.3390/polym16030366

**Published:** 2024-01-29

**Authors:** Eduardo Jara-Cornejo, Erick Peña-Bedón, Mahely Torres Moya, Sergio Espinoza-Torres, Maria D. P. T. Sotomayor, Gino Picasso, Juan C. Tuesta, Rosario López, Sabir Khan

**Affiliations:** 1 Technology of Materials for Environmental Remediation (TecMARA) Research Group, Faculty of Sciences, National University of Engineering, Lima 150128, Peru; ejjarac@uni.pe (E.J.-C.); erick.pena.b@uni.pe (E.P.-B.); mahely.torres.m@uni.pe (M.T.M.); sespinozat@uni.pe (S.E.-T.); gpicasso@uni.edu.pe (G.P.); 2 Institute of Chemistry, State University of São Paulo (UNESP), Araraquara 14801-970, SP, Brazil; m.sotomayor@unesp.br; 3 National Institute for Alternative Technologies of Detection, Toxicological Evaluation and Removal of Micropollutantans Radioactives (INCT-DATREM), Araraquara 14801-970, SP, Brazil; 4 Laboratorio de Biotecnología, Universidad Nacional Autónoma de Alto Amazonas, Calle Prolongación Libertad 1220, Yurimaguas 16501, Peru; jtuesta@unaaa.edu.pe; 5 Department of Natural Sciences, Mathematics, and Statistics, Federal Rural University of the Semi-Arid, Mossoro 59625-900, RN, Brazil; 6 Department of Exact Sciences and Technology, State University of Santa Cruz, Ilhéus 45662-900, BA, Brazil

**Keywords:** molecularly imprinted polymers (MIPs), curcumin, electrochemical sensor

## Abstract

Curcumin is a compound of great importance in the food industry due to its biological and pharmacological properties, which include being an antioxidant, anti-inflammatory, antibacterial, antiviral, and anticarcinogenic. This paper proposes the synthesis of an electrochemical sensor based on molecularly imprinted polymers (MIPs) and MWCNT by drop casting deposited on a glassy carbon electrode (GCE) for the selective quantification of curcumin in food samples. The synthesized compounds are characterized by Fourier transform infrared (IR), Brunauer–Emmett–Teller (BET), and electrochemical techniques such as cyclic voltammetry (CV) and differential pulse voltammetry (DPV). The optimal conditions for further experiments were determined by selecting these parameters. We examined three food products, commercial capsules, turmeric rhizomes, and commercial turmeric powder, employing both electrochemical and HPLC methods for the analysis. The electrochemical method revealed a limit of detection (LOD) value of 0.1365 µmol L^−1^, compared with the HPLC analysis, which gave a value of 3.55 µmol L^−1^. Furthermore, the MIP material demonstrated superior selectivity for the analyte compared to potential interferents. The recovery percentage, determined using the HPLC method, fell within the range of 87.5% to 102.6%

## 1. Introduction

Curcumin is a natural compound found in turmeric, which comes from the *Curcuma longa* plant. It is known for its potential health benefits, such as being anti-inflammatory, and antioxidant, and having positive effects on tumors and the brain. People use it in traditional cooking, and it is included in some health supplements. Because it is important in food and has health perks, it is essential to find good ways to quickly and accurately quantify its presence [1,2]. Analysis is crucial, as is the quantification of bioactive compounds in foods, to ensure food safety and evaluate its nutritional quality [3]. Curcumin is a polyphenolic compound present in turmeric, and it has been shown to have antioxidant, anti-inflammatory, and anticancer properties, among others. Its presence in foods is increasingly valued due to its health benefits [4]. However, achieving accurate and rapid quantification of curcumin in food samples remains challenging due to the complex nature of food matrices and interference from other compounds. Traditional methods such as high-performance liquid chromatography (HPLC) and spectrophotometry are expensive techniques, requiring specialized equipment and substantial time for analysis [5]. Hence, there is a necessity to create a device, like an electrochemical sensor utilizing molecularly imprinted polymers (MIPs), to quantify curcumin in food samples. MIPs are synthetic materials engineered to recognize and bind specific compounds, such as curcumin in this context. These sensors provide potential advantages, including rapid responses, enhanced sensitivity, and selectivity, as well as ease of miniaturization and cost-effective manufacturing [6].

However, despite the potential of MIP sensors, there are still challenges in their development and practical application. Some of the issues to address include optimizing the selectivity and sensitivity of the sensor, ensuring the stability and durability of the MIP material, dealing with interference from other food components, and validating the method in various food matrices [7,8].

In recent years, the analysis and quantification of bioactive compounds in foods have been the focus of numerous research efforts. In the specific case of curcumin, various analytical methods have been developed for its determination in food samples [9]. Among the traditional methods used are high-performance liquid chromatography (HPLC) and spectrophotometry. HPLC is a widely employed technique and is considered a reference in the analysis of bioactive compounds [10]. Recent studies have explored the application of this technique, enabling an efficient separation of components in a sample and an accurate quantification of curcumin [11]. Additionally, novel and straightforward methods, such as HPLC-UV, are also being developed [12].

However, this technique demands specialized and expensive equipment, as well as significant preparation and analysis time [13]. The uses of electrochemical sensors for the analysis of electroactive bioactive compounds in food have been investigated [14]. These sensors leverage the electrochemical properties of compounds, allowing for rapid and selective detection. Among the various designs of these sensitive and selective sensors are those based on graphene [15], carbon sensors fabricated with ruthenium-doped titanium dioxide nanoparticles coalesced (Ru-TiO_2_) with multi-walled carbon nanotubes (MWCNT) [16], and electrochemical super sandwich-type sensors [17].

Some researchers have determined the presence of curcumin using these electrochemical methods due to its ability to more readily donate electrons [18]. This technique offers advantages in terms of its simplicity and high sensitivity [19]. However, the lack of selectivity in electrochemical sensors for complex samples, such as food, can be overcome by coupling them with molecularly imprinted polymers (MIPs). MIPs act as receptors for electrochemical transduction in a cell, presenting a promising alternative [20,21].

The analytical methods mentioned earlier for curcumin detection lack selectivity, which is a limitation to their application. Molecularly imprinted polymers (MIPs) are being used for the detection and quantification of curcumin, providing increased sensitivity and selectivity. MIPs are synthetic materials designed to selectively recognize and bind a target compound, like curcumin. These materials are created through the polymerization of monomers in the presence of the target molecule or template; the interaction between them generates specific molecular recognition sites [22]. Subsequently, the template molecule is removed and eliminated through extraction. Therefore, MIPs can enhance a sensor’s ability to recognize the molecule corresponding to the template [23]. By using MIPs in electrochemical sensors, advantages such as a rapid response and improved sensitivity and selectivity, as well as easy miniaturization and low-cost manufacturing, can be achieved.

Over the past few years, several investigations have delved into the application of molecularly imprinted polymers (MIPs) for the detection of curcumin in diverse matrices, such as turmeric extracts, curcumin-enriched foods, and dietary supplements [19,24]. Molecular imprinting is a technique used to create specific analyte binding sites [25]. MIPs can be prepared by mixing functional monomers, crosslinkers, templates, and initiators in a solvent [18]. MIPs are synthetic adsorbents designed to selectively retain the target molecule regardless of the matrix’s complexity [26,27].

This material is valued not only for its high recovery efficiency but also for its ability to clean and enrich samples multiple times [24]. Due to these advantages, MIPs have been applied as solid materials in sensors, thanks to their high specificity, material durability, and stability [28]. Under optimal conditions, these sensors have shown promising results in terms of sensitivity and selectivity against some curcumin analogs [29].

Therefore, despite advances in the analysis of bioactive compounds in foods, the precise and rapid quantification of curcumin remains a challenge. Electrochemical sensors based on MIPs offer significant potential to overcome the limitations of traditional methods, but it is necessary to address issues such as the optimization of sensor selectivity and sensitivity, the stability and durability of the MIP material, interference from other food components, and the validation of the method in different food matrices.

This study proposes the synthesis of an electrochemical sensor employing molecularly imprinted polymers (MIPs) for the accurate quantification of curcumin in food samples. The motivation for this research arises from the crucial role curcumin plays in health, the need for an efficient and cost-effective analytical method, the benefits provided by MIPs as stable and selective elements, and their suitability for applications in the food industry.

## 2. Experimental Section

### 2.1. Chemicals and Solutions

The starting chemicals used for the preparation of the MIP were as follows: curcumin (CUR) from *Curcuma longa* (Turmeric) powder (C_21_H_20_O_6_, ≥66.5% (HPLC)) used as an analyte; acrylamide (AM) (C_3_H_5_NO, ≥99%, for electrophoresis) as a functional monomer; ethylene glycol dimethacrylate (EGDMA, C_10_H_14_O_4_, 98%) as a crosslinker; 4,4′-Azobis(4-cyanovaleric acid) (ABCVA, ≥98.0%) as a radical initiator; and acetonitrile (ACS reagent, ≥99.5%) as a porogenic solvent. For the MIP washing step, ethanol (EtOH, CH_3_CH_2_OH, for analysis EMPARTA^®^ ACS), methanol (MeOH, CH_3_OH, for liquid chromatography LiChrosolv^®^), and acetic acid (glacial) (CH_3_COOH, 100%) were used, all of which were purchased from Sigma Aldrich, St. Louis, MO, USA, as were phosphoric acid (Sigma Aldrich, 85%), boric acid (Sigma Aldrich, ACS reagent, ≥99.5%), and a multi-walled carbon nanotube (Sigma Aldrich, <5% modified with COOH).

For the preparation of the Britton–Robinson buffer, 0.62 mL of phosphoric acid, 0.57 mL of acetic acid, and 0.618 g of boric acid dissolved in 250 mL of water were used.

The interference compounds were resorcinol (RES), tannic acid (TAN), and L-ascorbic acid (L-AA); the colorant compounds were tartrazine (TRT) and sunset yellow (SY); and the environmental interfering compounds were sodium nitrate (NaNO_3_) and sodium chloride (NaCl). All these reagents came from Sigma Aldrich, ACS reagent, ≥99.5%.

### 2.2. Instrumentation

Fourier transform infrared (FTIR) spectra were recorded on Bruker FTIR–ALPHA II Spectrometer (Billerica, MA, USA). The samples were dried at room temperature before the analysis. The spectra were scanned over the range of 4000 to 400 cm^−1^ in the ATR mode to identify functional groups in the structure of the polymers.

Electrochemical analyses were carried out employing the three-electrode setup, comprising a glassy carbon electrode (with a diameter of 3 mm) serving as the working electrode, an Ag/AgCl reference electrode immersed in a 3 mol L^−1^ of KCl solution, and a platinum wire electrode as the auxiliary electrode. These materials were obtained from Microtube, Brazil, Araraquara. The measurements were conducted at room temperature (25 °C) using an AUTO LAB PGSTAT 100 potentiostat–galvanostat, controlled by NOVA 2.1.1 software. Analytical-grade reagents were utilized for all the experiments, and the solutions were prepared with deionized water sourced from Milli-Q Direct-0.3 (Millipore, Billerica, MA, USA). The electrochemical profiles of curcumin were obtained using differential pulse voltammetry in 0.1 mol L^−1^ of Britton–Robinson buffer solution (pH 2.0) in a potential range of 0.1 to 0.9 V. For the analyte detection tests, an adequate volume of the standard solution of 100 µmol L^−1^ of curcumin dissolved in methanol was added. The parameters in DPV were studied to obtain the best oxidation signal, such as the contact time, 5 mV step potential, 0.025 V amplitude, 0.05 s modulation time, and 0.1 s interval time. Meanwhile, the CV analyses were performed at a scan rate (υ) of 50 mV s^−1^, a potential range of 0.1 to 0.9 V, and a potential step of 2.44 mV.

The chromatographic analysis was carried out with the Vanquish Thermo Scientific HPLC equipment (Waltham, MA, USA), composed of a model VF-P20 quaternary pump, a VF-A10 autosampler, a VH-C10 column oven, and a VF-D11A diode array detector (DAD). All modules were controlled with Xcalibur software version 4.2.47. All separations were performed on an RP C18 column (100 mm × 2.1 mm ID, 3 µm particle). Each sample injection was 2 µL at a flow rate of 1 mL min^−1^ of the mobile phase with a temperature of 15 °C; for the sample analysis, the gradient was prolonged up to 25 min by maintaining the gradient slope of the mobile phase gradient in A (0.1% acetic ac. in water) at 40% and in B (0.1% acetic ac. in acetonitrile) at 60%.

### 2.3. Sample Collection and Preparation

For the treatment of the curcumin samples, three food products were obtained, including commercial capsules, turmeric rhizomes obtained from the city of Yurimaguas, and commercial turmeric powder.

For the treatment of the three samples, 20 mg, 100 mg, and 25 mg, respectively, were weighed and dissolved in 100 mL of methanol each. The solution was then sonicated for 30 min, followed by 15 min of centrifugation, and finally filtered using a membrane with a pore size of 20 µm.

### 2.4. Synthesis of Molecularly Imprinted Polymer (MIP) and Molecularly Non-Imprinted Polymer (NIP) by Precipitation Method

**Figure 1:**
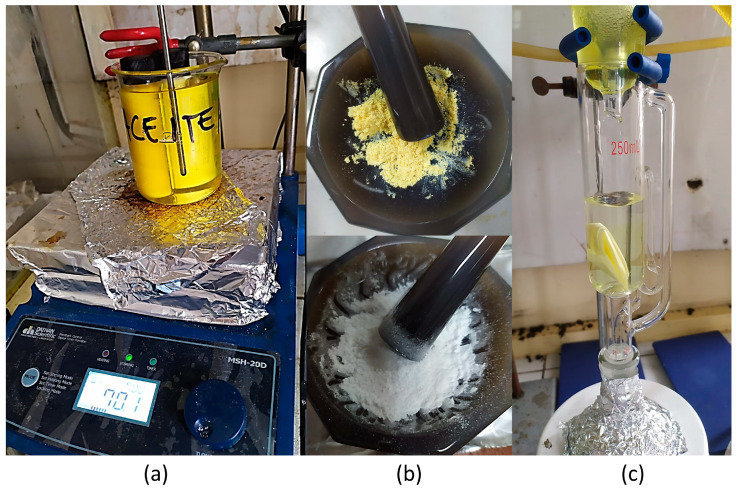
(**a**) Molecular imprinted polymer (MIP) synthesis; (**b**) MIPs and NIPs before washing with Soxhlet system; (**c**) washing of MIPs with Soxhlet.

The MIPs were synthesized through the bulk polymerization method (Figure 1); this method consists of the formation of a pre-polymerization complex by stirring for 2 h the template (curcumin), functional monomer (acrylamide), and porogenic solvent (acetonitrile) in a sealed flask to promote the curcumin–acrylamide interaction. Next, the mixture is bubbled with N_2_ gas for 10 min, the crosslinker (EGDMA) is added, and N_2_ bubbling continues for another 10 min. Finally, the radical initiator is added, and immediately, the sealed flask is placed in an oil bath at 70 °C for 2 h. The molar ratio of curcumin, acrylamide, and EDGMA was 1:4:50, respectively (Table 1). Particles without a molecularly imprinted surface (NIP) were also prepared under the same conditions but without the presence of curcumin.

For the extraction of curcumin from the MIPs and the formation of cavities, a Soxhlet extraction system was used with mixtures of methanol and acetic acid (9:1 y 7:3 *v*/*v*) for 72 h. The main intense absorption band of curcumin is at 430 nm, so the wash mixtures were measured in that region using UV-Vis spectroscopy (Thermo Scientific GENESYS 10S Series, Waltham, MA, USA) to monitor the extraction of curcumin molecules from the polymer surface. The polymers were dried at 60 °C overnight and sieved to obtain sizes of approximately 250 nm.

### 2.5. Modification of the Glassy Carbon Electrode

The preparation of the modified electrode was carried out through an electro-chemical treatment where a suspension was prepared with 2 mg of polymers (MIPs or NIPs) and 2 mg of MWCNT in 2 mL of DMF, followed by 60 min of sonication and 30 min of stirring. Subsequently, 5 µL of this suspension was deposited on the surface of the electrode by drop casting. Finally, an IR lamp was used for the complete evaporation of the solvent until drying.

### 2.6. Application of Modified Sensor to Real Samples

For the HPLC analyses, 100 mg of curcumin standard was dissolved in 100 mL of HPLC-grade methanol to obtain a curcumin stock solution of 1000 µmol L^−1^, which was used to obtain a calibration curve whose linear range was 2.7–81.44 µmol L^−1^ and whose medium was HPLC-grade methanol. In the treatment of the curcumin samples, three food products were acquired, namely commercial capsules, roots from the city of Yurimaguas, and commercially available powder. For the treatment of the root, powder, and capsule samples, 20, 100, and 25 mg were weighed, respectively, and each was dissolved in 100 mL of methanol. Then, they were sonicated for 30 min, centrifuged for 15 min, and then filtered using a membrane with a pore size of 20 µm. For both the HPLC technique and the electrochemical tests, the standard addition technique was used in order to reduce any possible matrix effect during the electroanalytical analysis, so known concentrations of a curcumin standard were added: 0, 13.5, and 27 µmol L^−1^ for each test.

## 3. Results and Discussion

### 3.1. Textural Morphological and FTIR Characterization of the MIPs and NIPs

**Figure 2:**
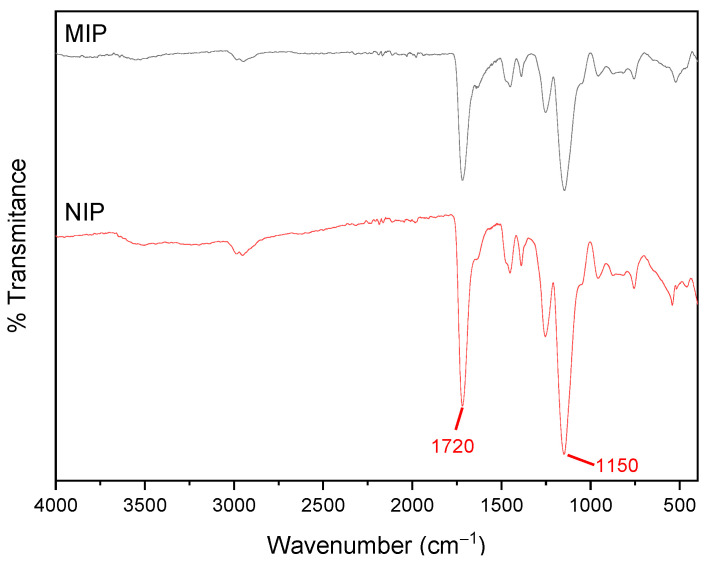
FTIR spectra of MIPs and NIPs after washed using Soxhlet system.

The FTIR spectra, as shown in Figure 2, exhibit comparable features for both the MIPs and NIPs, emphasizing distinctive peaks at 1720 cm^−1^ and 1150 cm^−1^. These peaks are attributed to the C=O (carbonyl) and C–O (ether) bonds, respectively. Notably, these bonds are derived from the ester group present in the crosslinker monomer, constituting a significant portion exceeding 90% of the polymer composition. This similarity in spectral characteristics underscores the close resemblance in the chemical makeup of both molecularly imprinted polymers (MIPs) and non-imprinted polymers (NIPs).

**Figure 3:**
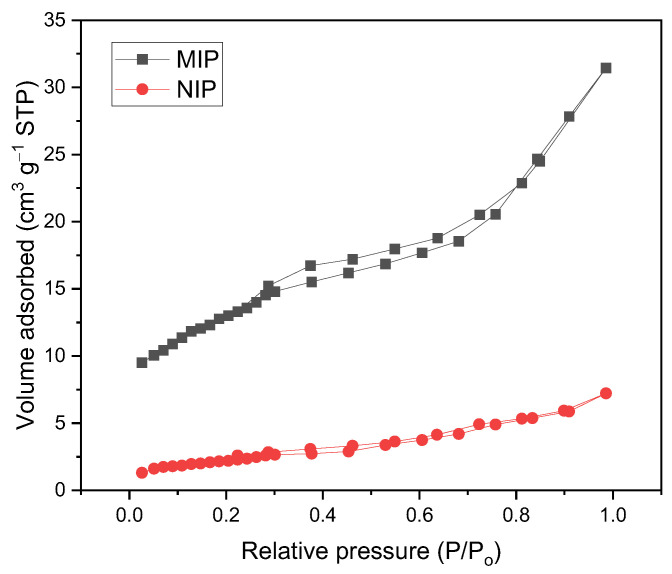
N_2_ adsorption–desorption isotherms using the BET method for MIPs and NIPs.

The BET isotherms of the MIPs and NIPs are type IV, as shown in Figure 3, which is typical of mesoporous solids, and they also present type H_3_ hysteresis. Table 2 shows the BET surface area of an MIP (48.9 m^2^ g^−1^), which is four times greater compared to that of an NIP (10.9 m^2^ g^−1^); likewise, the MIPs presented a greater mesoporous surface (40.8 m^2^ g^−1^) and pore diameters less than 50 nm, which classifies them as mesopores. This increase in surface area may be due to the presence of cavities in the printed polymer and the possible increase in active sites on the surface of the material [30].

### 3.2. Electrochemical Behavior of Curcumin

**Figure 4:**
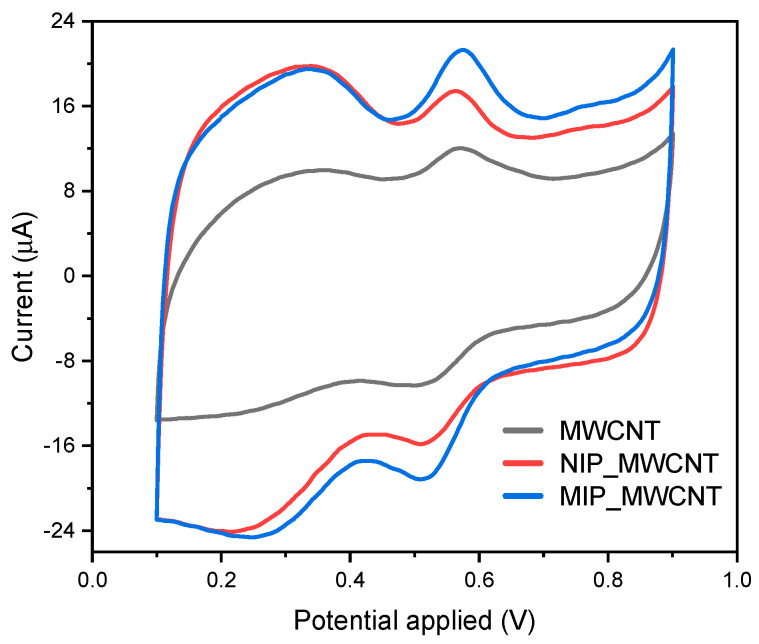
CV profile of 25 µmol L^−1^ of curcumin in a pH 2 BR buffer from modified electrodes.

The electrochemical oxidation behavior of curcumin was evaluated. Figure 4 shows the first CV cycle in a potential range of 0.1 to 0.9 V vs. Ag/AgCl at a scan rate of 50 mV s^−1^. The profile presented shows that the redox mechanism is reversible at the apparent standard potential, E^0^ = (E_pa_ + E_pc_)/2, where E_pa_ and E_pc_ are the potentials of the anodic and cathodic peaks, 540.6 mV vs. Ag/AgCl, with a separation potential, ΔE, of around 63 mV. According to what is reported in the literature, an adsorption process of curcumin occurs that, through an electrochemical process with the influence of acidic pH, results in the 2-methoxyphenol derivative of curcumin becoming a curcumin derivative similar to 1,2-dihydroxybenzene [31].

This adsorption of curcumin is more intensified by the presence of the cavities on the printed polymer (Figure 4; red vs. blue curve); in response to a material with more electroactive sites, a more intense oxidation peak is observed. So, what is shown in Figure 4 is more in line with what was expressed above, i.e., an electrochemical reaction that involves the mobility of four electrons to obtain an electrooxidized curcumin compound [32].

**Figure 5:**
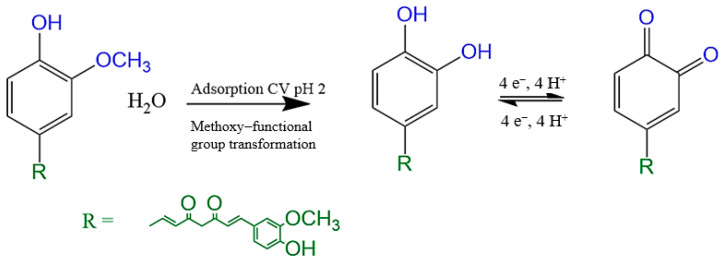
The proposed mechanism for curcumin oxidation.

**Figure 6:**
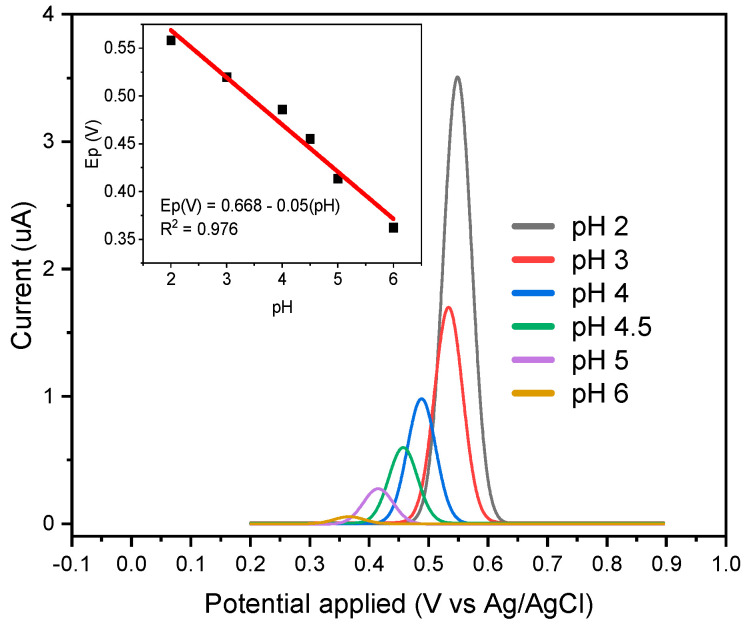
Differential pulse voltammograms obtained from the application of 0.4 μmol L^−1^ of curcumin at different pHs.

The impact of the solution’s pH was investigated, as shown in Figure 5. Differential pulse voltammetry measurements were carried out in aqueous Britton–Robinson (BR) buffer solutions with different pH values in a range of 2 to 6, using a scan speed of 50 mV s^−1^. A maximum pH value of 6 was considered because at higher pHs, curcumin degrades and the compound greatly loses its affinity with the cavities of the printed polymer. The results indicate that the redox potential responsible for the direct transfer of electrons from the adsorbed curcumin experiences a negative shift with increasing pH. The graphical representation of the formal potential versus pH, as illustrated in Figure 6, shows a linear relationship, indicating that a proton transfer reaction accompanies the electron transfer between the immobilized curcumin and the electrode. The value of the slope obtained is −50 mV pH^−1^, which is close to the expected value of −58 mV pH^−1^ for a reversible electron transfer process associated with a proton reaction, according to the Nernst equation [32].

**Figure 7:**
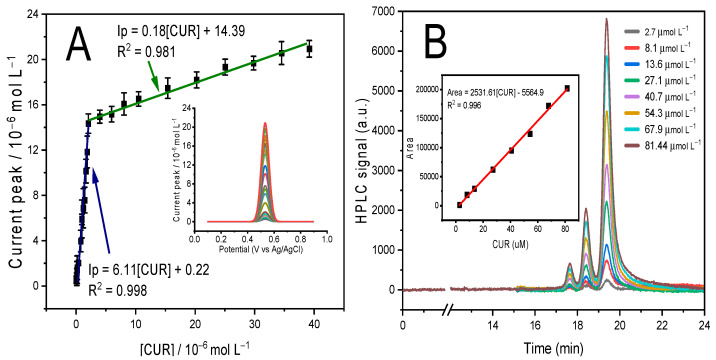
(**A**) Calibration curve for the detection of curcumin based on the application of MIP/MWCNT/GCE in BR buffer with pH 2. Inset: DPV analysis. (**B**) Calibration curve for the HPLC method.

Figure 7A shows the calibration curve for the proposed sensor, achieving Ip_1_ = 6.11 [CUR] + 0.22 with R^2^ = 0.998 in a concentration range of 0.04 to 2 μmol L^−1^ and Ip_2_ = 0.18 [CUR] + 14.39 with R^2^ = 0.981 in a concentration range of 2 to 40 μmol L^−1^. The presence of these two concentration ranges is due to the affinity of the curcumin-printed cavity of the polymer. So, at low concentrations of the analyte, it happens that the analyte tends to be adsorbed with high affinity by the polymer cavity; however, when increasing the concentration of the analyte to values greater than 2 μmol L^−1^, a saturation of the molecules of the analyte may occur, and this repulsion between the same molecules inhibits the electrostatic affinity of the printed polymer cavity, decreasing the sensitivity of the sensor [33]. On the other hand, Figure 7B shows the calibration curve for the HPLC analysis with an LOD of 3.55 μmol L^−1^, while for the proposed electrochemical sensor method, the LOD is 0.037 μmol L^−1^, which shows the advantage of the proposed sensor since the sensor is capable of detecting 95 times more than HPLC. This limit of detection (LOD) was calculated as 3.3 × SB/m, where SB is the standard deviation of the blank sample and m corresponds to its slope.

### 3.3. Analysis of Selectivity

**Figure 8:**
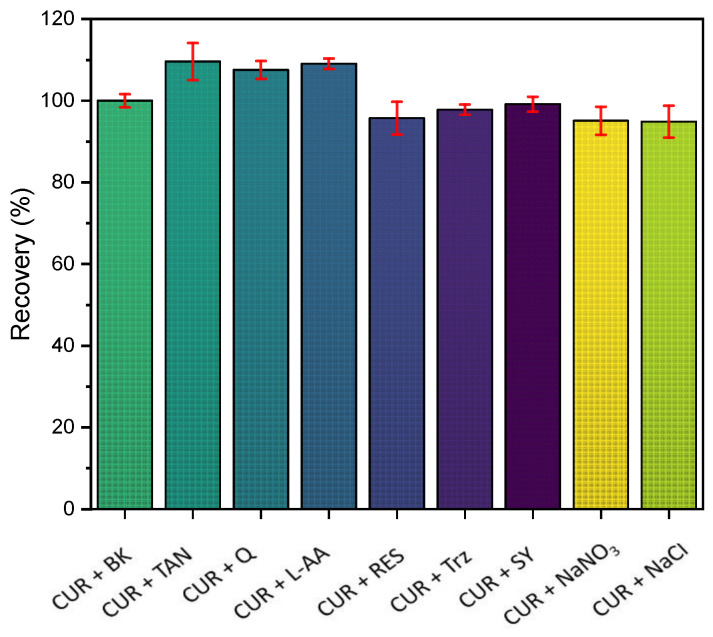
Interference study carried out using MIP/MWCNT/GCE.

The selectivity study of a sensor is a scientific investigation that delves into how well a sensor can distinguish and respond specifically to a certain target substance, known as an analyte. This study is particularly crucial in fields such as analytical chemistry and sensor technology, where the accurate and reliable detection of specific substances is essential in the presence of potential interferences. In evaluating the potential interference in the detection of curcumin (the target compound) using MIPs, we performed electrochemical experiments with compounds that share structural similarities to curcumin while being uniquely distinct from the target molecule. Resorcinol (RES), tannic acid (TAN), L-ascorbic acid (L-AA), tartrazine (TRT), sunset yellow (SY), sodium nitrate (NaNO_3_), and sodium chloride (NaCl) were used in the selectivity test. No significant changes were observed even when the molar ratios of these compounds and curcumin were equal. The detailed results, presented in Figure 8, show a visual representation of the experiment outcomes. Significantly, the proposed electrochemical biomimetic sensor showed robust selectivity, proving its proficiency in accurately distinguishing curcumin from other compounds. This noteworthy selectivity was evident during rigorous testing in the presence of two compounds with very similar chemical structures to curcumin, with some differences in structure as well, underscoring the sensor’s promising potential for precise detection in complex environments.

### 3.4. Repeatability Study

**Figure 9:**
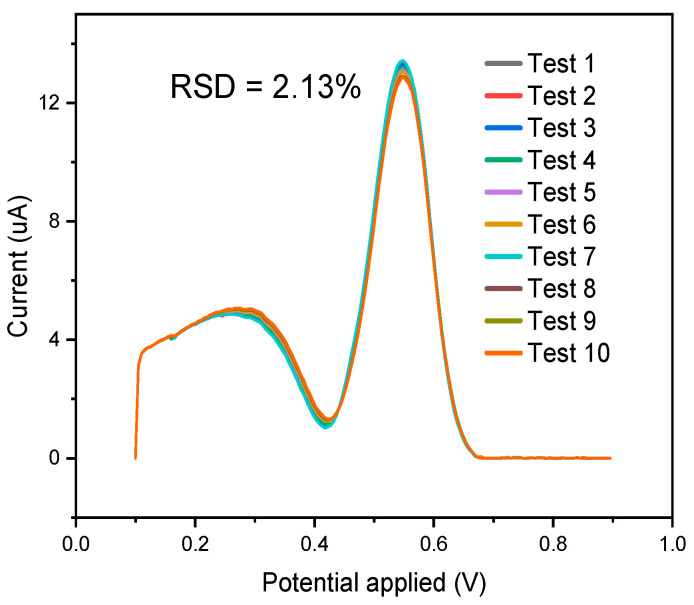
Differential pulse voltammograms obtained from the application of 0.4 μmol L^−1^ of curcumin; *n* = 10 replicates.

Repeatability is a measure of how consistently a sensor or system produces the same results when subjected to the same conditions over multiple trials. In the context of the MIP sensor, it refers to the ability of the sensor to provide reproducible and reliable measurements. The repeatability of the sensor was evaluated based on a 10-time analysis, as shown in Figure 9 below. This indicates that a systematic examination of the sensor’s repeatability was conducted through a series of ten analyses, and relative standard deviation (RSD) results were obtained, revealing a value of about 2.13%.

### 3.5. Applications to Real Samples

**Figure 10:**
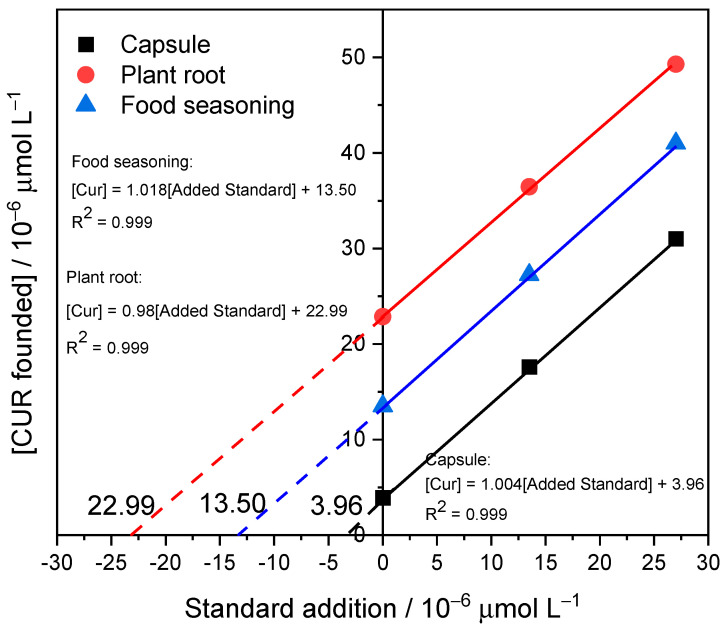
Quantification curve by standard addition method of real samples: capsule, plant root, and food seasoning.

The efficacy of the proposed electrochemical sensor was examined using three genuine samples. Commercial capsules, turmeric rhizomes (plant root), and commercial turmeric powder (food seasoning) were intentionally spiked with varying concentrations at two distinct levels, 13.5 and 27 µmol L^−1^, as shown in Figure 10. The results presented in Table 3 suggest that both HPLC and the proposed electrochemical methods exhibit good recovery after spiking with a known concentration. Furthermore, the percent error is less than 4%, and the percent recovery falls within the range of 98.7% to 103.98%, indicating the reliability and accuracy of the analytical techniques employed. Table 4 shows a comparative analysis of the proposed method with the existing literature. The table provides a comprehensive overview of key parameters like real samples, method, percentage recovery, and the limit of detection.

## 4. Conclusions

In this study, a molecularly imprinted polymer (MIP) that is selective towards curcumin was successfully synthesized. The application of molecularly imprinted polymers as recognition materials proved effective for the quantification of curcumin in real samples, with the limit of detection (LOD) value using the electrochemical method reported at 0.1365, while for the HPLC method, it was 3.55 µmol L^−1^. Additionally, the MIP material exhibited higher selectivity towards the analyte compared to some of the interfering compounds. The percentage of recovery was found in the range of 87.5 to 102.6% using the HPLC method.

Additionally, the synthesized material was tested through the analysis of real samples, including three different types of curcumin-containing products obtained from a local market in Peru, to assess the efficacy and practical applicability of the molecularly imprinted polymers in real samples. Therefore, the proposed approach holds the potential to enhance the efficiency of time-consuming methods employed for the quantification of curcumin in water samples.

## Figures and Tables

**Table 1:** Amounts of reagents used for the synthesis of MIPs.

	Amounts	Concentration (mM)	Molar Ratio	
Curcumin	0.1 mmol	2.5	%T	
Acrylamide	0.4 mmol	10.0	(Acrylamide (g) + EGDMA(g))/V(mL)	2.55
EGDMA	5.0 mmol	125.0	%C	
ABCVA	100 mg	-	EGDMA/(Acrylamide + EGDMA)	0.926
Acetonitrile	40 mL	-	Template:(Acrylamide + EGDMA)	1:54

**Table 2:** BET surface area and porosity obtained for MIPs and NIPs.

Polymer	BET Surface Area (m^2^ g^−1^)	Micropore Area (m^2^ g^−1^)	Mesopore Area (m^2^ g^−1^)	Average Pore Diameter (nm)
MIP	48.9	8.1	40.8	40.1
NIP	10.9	1.5	9.4	43.0

**Table 3:** Results obtained from the analysis of the capsule, plant root, and food seasoning samples.

Sample	Added Concentration (µmol L^−1^)	HPLC Method	Electrochemical Method	% Recovery of Proposed Method *	%Error **
		Sample Concentration	Sample Concentration		
		(µmol L^−1^)	(µmol L^−1^)		
Capsule	0	3.81	3.92	103.98	3.53
	13.5	17.4	17.6		
	27	30.89	31.03		
Plant root	0	22.92	22.87	100.33	−2.18
	13.5	37.09	36.46		
	27	47.73	49.29		
Food seasoning	0	13.67	13.51	98.78	−1.41
	13.5	27.32	27.24		
	27	40.81	41.01		

* %Recovery = $\frac{\left[Intercept\;of\;the\;calibration\;curve\;of\;the\;proposed\;method\right]}{\left[HPLC\;Initial\;Concentration\right]}\times 100$; ** % Error = $\frac{\left[Intercept\;of\;the\;proposed\;method\right]\;-\;[Intercept\;of\;the\;HPLC\;method]}{\left[Intercept\;of\;the\;calibration\;curve\;of\;the\;HPLC\;method\right]}\times 100.$

**Table 4:** Summary of results of other research for the development of a sensor for curcumin quantification.

Material	Analyte/ Real Sample	Method	LOD/ %Recovery	Ref.
MIP-CPE Sensor	Curcumin/ Curcuma powder and cookies	CV	10.1 nmol L^−1^ 90.77~105.7%	[34]
PAA-MIP/ G Sensor	Curcumin/ Turmeric powder and capsules	DPV	0.04 µmol L^−1^ >99.00%	[28]
GCE/CuCo_2_O_4_/N-CNTs/P-GO/MIP (L-Cys)	Curcumin/ Serum	DPV	30 nmol L^−1^ 80.00–110.87%	[23]
MIP-CPE Sensor	Curcumin/ Turmeric powder	CV	0.02 µmol L^−1^ 1–100 µmol L^−1^	[35]
CUR-MIP/GCE	Curcumin/ Turmeric extract	DPV	5.0 nmol L^−1^ 0.1–2.0 mmol L^−1^	[36]
MIP/MWCNT/GCE Sensor	Curcumin/ Capsule, plant root, and food seasoning	CV/DPV	0.136 µmol L^−1^ 87.5–102.6%	This work

## Data Availability

Data are contained within the article.
